# Measurement of Occupational Eye and Thyroid Radiation Doses in Pediatric Interventional Cardiologists at a Tertiary Hospital

**DOI:** 10.7759/cureus.44894

**Published:** 2023-09-08

**Authors:** Mawya Khafaji, Tariq M Ashour, Naif F Mozahim, Abdullah Tashkandi, Basil Alqarni, Abdulrahman A Malaikah, Abdulaziz K Bagabas, Abdullah A Alwasabi, Sarah K Albahiti

**Affiliations:** 1 Radiology Department, King Abdulaziz University Faculty of Medicine, Jeddah, SAU; 2 Faculty of Medicine, King Abdulaziz University, Jeddah, SAU

**Keywords:** thyroid dose, eye lens dose, staff exposure, intervention pediatric cardiology, occupational radiation dose, cardiac catheterization

## Abstract

Background

Advances in imaging techniques have led to increased utilization of imaging devices in catheterization laboratories. Invasive surgical procedures for cardiac disorders have been largely replaced by fluoroscopic cardiac catheterization. With this increase, concerns and risks associated with exposure to ionizing radiation among interventional cardiologists are growing. This study aims to measure and compare the occupational doses to the eye lens and thyroid of pediatric interventional cardiologists during different procedures in the catheterization laboratory and its significance.

Methodology

In this study, cardiologists wore bandanas with attached dosimeters to measure the absorbed doses to the eyes and thyroid gland. The dosimeters were collected for reading. The procedure types were also collected. In addition, the total fluoroscopy time and tube voltage of the biplane machine were measured. SPSS version 23 (IBM Corp., Armonk, NY, USA) was used to analyze the data. The characteristics of the study sample were described using simple counts and percentages, whereas means and standard deviations were used for continuous variables. Statistical significance was set at p-values <0.05.

Results

A total of 93 procedures were evaluated. The mean absorbed doses for all 93 procedures in both eyes and thyroid were 0.09 mGy and 0.08 mGy, respectively. A significant difference was found between the left and right eye measurements (p = 0.034), with higher doses administered to the left eye. However, no significant difference was observed between the right and left thyroid doses (p = 0.281). Significant correlations were found between the eye and thyroid doses and the procedure type (p = 0.02 and p = 0.009, respectively).

Conclusions

A significant amount of radiation was measured in the measurements of both organs. In addition, radiation dose measurements varied between different types of procedures. Our current results indicate the importance and necessity of applying the radiation protection concept of dose optimization.

## Introduction

Advances in imaging techniques and the use of X-rays have led to increased utilization of imaging devices in catheterization laboratories. Ionizing radiation is implemented using fluoroscopy, which is widely used during cardiac catheterization procedures in the pediatric population [1].

Cardiac catheterization is used to diagnose and treat certain cardiovascular disorders and conditions. Invasive surgical procedures for cardiac disorders have been largely replaced by fluoroscopic cardiac catheterization [2]. Over the past two decades, cardiac catheterization procedures have increased significantly. However, with this increase, concerns and risks associated with exposure to ionizing radiation among medical staff involved in these procedures are growing [3].

Cardiologists and technical workers in catheterization laboratories face specific occupational health risks. The most hazardous aspect of the job is long-term radiation exposure, as cardiologists and their teams accumulate considerable amounts of radiation throughout their professional lives, ranging from 50 to 200 mGy. This level of exposure is equivalent to having between 2,500 and 10,000 chest X-rays and is projected to result in a one in 100 excess cancer risk throughout their careers. Furthermore, persistent exposure to occupational radiation has been associated with an elevated risk of developing cataracts, cancers, and other conditions such as premature vascular and neurocognitive aging [4].

Typically, when performing interventional procedures, cardiologists must be in close proximity to the radiation beams. In the case of pediatric cardiac interventional procedures, this proximity is even more pronounced, as the cardiologist has to be close to the patient compared to cardiologists performing these procedures on adult patients [5].

In 2017, a study was conducted in Japan to measure the occupational exposure of medical personnel performing interventional cardiology procedures using a new direct eye dosimeter. The researchers measured the eye dose received by physicians and nurses working in a catheterization laboratory over six months. The results showed that cardiologists and nurses who did not wear leaded glasses had higher eye doses than those who did. These findings suggest that interventional cardiologists are at greater risk of radiation exposure than other healthcare workers in this setting [6].

In 2016, a study conducted in Chile aimed to establish a correlation between the age groups of pediatric patients and radiation exposure experienced by medical staff. The research findings indicated that for the four age groups of patients and procedures, the scattered dose to the lower extremities of the cardiologist ranged from 1 to 28 mGy (for patients under one year), 6 to 58 mGy (for those under five years), 13 to 155 mGy (for those under 10 years), and 29 to 375 mGy (for those under 15 years) [7].

In 2012 and 2013, two studies in Spain highlighted that the eye lens is more sensitive to radiation than previously considered. Furthermore, radiation-induced cataract formation indicates that the actual dose limit for the eye lens may be too high [8,9]. In 2011, a French study involving 106 interventional cardiologists and 99 non-exposed subjects revealed a higher incidence of posterior subcapsular lens opacity among interventional cardiologists. The incidence rate was significantly higher among interventional cardiologists than in the non-exposed group (17% vs. 5%, p = 0.006) [10].

The thyroid gland is sensitive to ionizing radiation; therefore, safeguarding it during medical procedures is crucial [11]. Radiation exposure can potentially cause thyroid cancer, with age and the radiation dose to the thyroid being significant risk factors [11]. Although the exact risk of scattered radiation to the thyroid gland remains unknown, studies have indicated that a cumulative dose of 0.065 mGy per procedure increases the long-term risk of thyroid cancer [12].

Radiation-related adverse effects can be reduced by lowering the patient’s dose, increasing the distance, and using shielding. However, this comes at the expense of the image quality produced. Radiation-protective lead aprons are most effective in reducing the operator dose and have been adopted as the standard protective practice in most clinical settings. The use of leaded glasses can reduce radiation exposure to the eyes by as much as 98%, and incorporating side panels into these glasses can further enhance their protective capabilities. To minimize the risk of radiation-induced thyroid cancer during laboratory catheterization procedures, thyroid shields are recommended among the most effective methods for reducing exposure. To ensure maximum effectiveness, it is crucial to minimize gaps between the thyroid collar shield and the lead apron [13,14].

Most studies have measured physicians’ exposure to adult procedures. However, data on occupational doses in pediatric interventions are still lacking. This study aims to measure and compare radiation exposure to the eye lens and thyroid in pediatric interventional cardiologists.

## Materials and methods

Study design

This prospective cohort study was conducted in December 2021 at the Catheterization Laboratory of King Abdulaziz University Hospital in Jeddah, Saudi Arabia. The study was approved by the hospital’s ethics committee (reference number: 347-21).

Dosimeters

**Figure 1:**
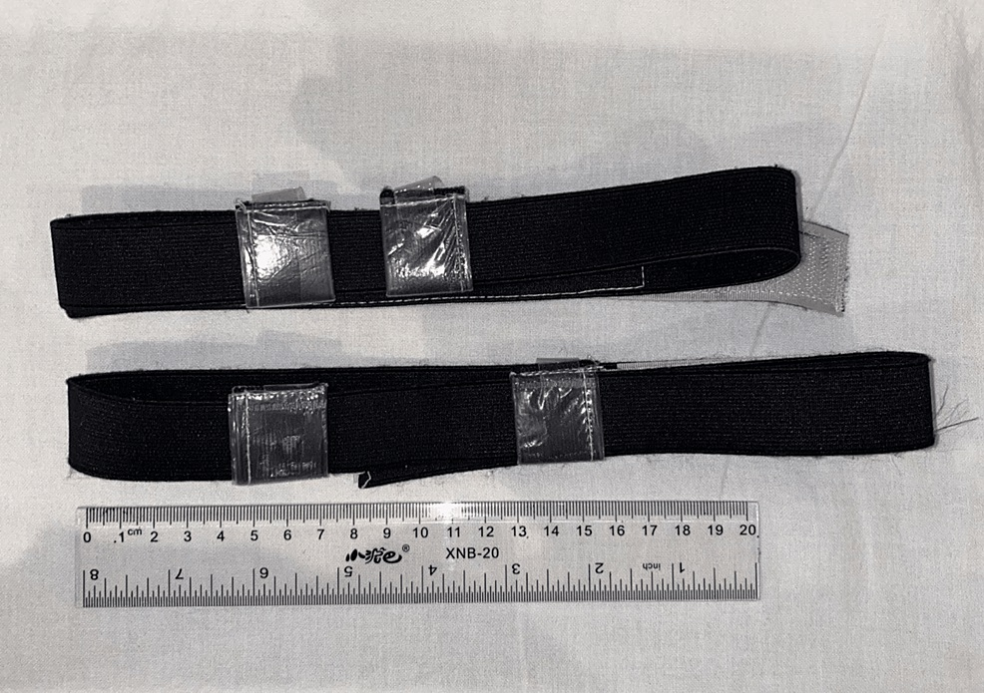
Custom-made bandanas for measuring eye and thyroid doses.

NanoDots (nano-Dot®, Landauer, Inc., Glenwood, IL) are optically stimulated luminescence dosimeters (OSLDs). Four dosimeters were used for each procedure. These dosimeters, which measured 10 x 10 x 2 mm, were placed inside a light-plastic casing and attached to a custom-made bandana (Figure 1). MicroStar® reader (Landauer, Inc., Vélizy-Villacoublay, France) was then utilized to measure the absorbed dose. The process of annealing the nanodots serves the crucial purpose of eliminating any radiation present in the nanoDotTM OSLDs. This dose may have been acquired during storage because of previous radiation exposure. A microStar Pocket Annealer (model number 12040765) was utilized to achieve this. This compact device could efficiently clear doses of up to 0.1 Gy on the nanoDotTM OSLDs. It is worth noting that the microStar pack comes with a Pocket Annealer as a standard inclusion, providing a comprehensive solution.

Equipment parameters

All procedures were performed using a biplane fluoroscopy machine (GE Innova Igs 620). This system uses flat-panel detectors to capture high-resolution X-ray images and fluoroscopic sequences. It offers both two-dimensional and three-dimensional imaging capabilities, allowing for detailed visualization of the anatomy and the guidance of medical instruments. It is equipped with a square flat-panel digital detector measuring 20.5 x 20.5 cm. It typically features a C-shaped gantry that can be maneuvered around the patient. This flexibility allows for various angles and positions during imaging and procedures. The machines had two planes, A and B, with a 60-120 kVp tube voltage range and current levels ranging from 35 to 60 mAs. Four pediatric cardiologists performed all cardiac procedures. Ceiling-mounted lead shields were used in all procedures.

Procedures

The various procedures were classified into the following three categories: diagnostic, non-complicated, and complicated. This categorization was performed according to an assessment by a pediatric interventional cardiologist of the presented cases. Complicated procedures involve intricate techniques, require advanced skills, and demand precision in execution compared to non-complicated procedures. The non-complicated procedures included right or left pulmonary artery stenting, atrial septal defect closure, patent ductus arteriosus/ventricular septal defect closure, right/left pulmonary artery ballooning, and pulmonary valvuloplasty. Furthermore, complicated procedures include patent ductus arteriosus stenting, venovenous malformation coiling, and coarctation of the aorta. The inclusion criterion was any procedure performed by a pediatric cardiologist during the specified timeframe in which NanoDots were available, even if certain procedures lacked complete dosage measurements.

Data collection method and data entry

Four pediatric cardiologists wearing lead aprons and thyroid collars participated in this study. In addition, ceiling-mounted lead shields were used. Each participant was provided with four dosimeters during each procedure; two dosimeters were worn above each eye to measure eye doses, and another two dosimeters, attached to a custom-made bandana, were placed at neck level anteriorly under the thyroid collars to assess thyroid doses. These dosimeters had to be held in place for the entire procedure duration and were removed immediately after completion to ensure accurate dose readings. Data on the demographics of each procedure (patient age, medical record number, and date of procedure) and procedure type (diagnostic, non-complicated, and complicated) were collected. Procedures were classified according to age group (less than one year old, between one and five years old, between 5 and 10 years old, between 10 and 14 years old, and more than 14 years old). The cardiologist obtained the procedure, eye, and thyroid dose-related data after reading the NanoDots and X-ray machine parameters (kVp, total acquisition time, dose area product total, and total fluorotime).

Statistical analysis

SPSS version 23 (IBM Corp., Armonk, NY, USA) was used to analyze the data. The characteristics of the study sample were described using simple counts and percentages, whereas means and standard deviations were used for continuous variables. A paired-sample t-test was used to compare the means of two variables within a group. One-way analysis of variance was used to compare more than two groups with the least significant difference as a post-hoc test for normally distributed data and Games-Howell for non-normally distributed data. Pearson’s correlation coefficient was used to correlate means, and the Kruskal-Wallis test was used to test several independent sample procedures for cases with non-normal distribution. Statistical significance was set at p-values <0.05.

## Results

This study included 93 cases that were categorized into three types. Among the 93 procedures, 39.1% were diagnostic, 41.3% were uncomplicated, 19.6% were complicated, and one remained uncategorized due to the absence of pertinent information regarding the procedures’ specifics. The age groups were classified as follows: 36.6% were one year old, 35.5% were 1-5 years old, 18.3% were 6-10 years old, 6.5% were 11-14 years old, and 3.2% were older than 14 years. The average age of the patients was 3.84 years, and three patients were older than 14 years. The mean absorbed radiation dose to both eyes was 0.09 mGy (standard deviation of ±0.05 mGy), and the maximum dose was 0.28 mGy. As for thyroid dose, the mean was 0.08 mGy (with a standard deviation of ±0.06) and a maximum dose of 0.38 mGy (Table 1).

**Table 1:** Characteristics of study samples.

Variables	N	Minimum	Maximum	Mean	SD	Median
Right eye dose (mGy)	89	0.03	0.35	0.08	0.1	0.07
Left eye dose (mGy)	89	0.04	0.41	0.10	0.1	0.08
Right thyroid dose (mGy)	88	0.02	0.63	0.08	0.1	0.06
Left thyroid dose (mGy)	88	0.03	0.42	0.08	0.1	0.06
Eye dose (mGy)	89	0.04	0.28	0.09	0.05	0.08
Thyroid dose (mGy)	88	0.03	0.38	0.08	0.06	0.06
Age of patient (years)	93	0.02	28.00	3.84	5.0	1.75
			Count		%	
Total			93		100.0	
Case	Not complicated		38		41.3	
	Diagnostic		36		39.1	
	Complicated		18		19.6	
	Non-categorized		1			
Age of patient	<1 year		34		36.6	
	1–5 years		33		35.5	
	6–10 years		17		18.3	
	11–14 years		6		6.5	
	>14 years		3		3.2	

The rationale behind the varying N values (89 for eye doses and 88 for thyroid doses) in the table can be attributed to certain technical issues or circumstances in which the measurements of eye or thyroid doses were not feasible during certain procedures. These challenges may have arisen because of technical limitations regarding the calibration of nanodots or the utilization of equipment, such as bandanas, by the physicians involved.

Pediatric cardiac procedures were performed using biplane radiography (planes A and B). For comparison, a limited set of 65 procedures was acquired from the digital system of the catheterization laboratory. The remaining procedures were not attainable due to unavailability. For plane A, the mean kVp was 63.49 (with a standard deviation of ±3.5), the mean total acquisition time was 45.82 seconds (with a standard deviation of ±33.2), and the mean dose area product total was 9.33 Gy.cm^2^ (with a standard deviation of ±19.9). For plane B, the mean kVp was 64.29 (with a standard deviation of ±6.1), the mean total acquisition time was 46.95 seconds (with a standard deviation of ±34.2), and the mean dose area product total was 6.05 Gy.cm^2^ (with a standard deviation of ±18.8). The mean total fluoro-time was 1,270.23 seconds (Table 2).

**Table 2:** Biplane parameters during procedures.

Variables		N	Minimum	Maximum	Mean	SD	Median
Plane A	kVp	65	60.00	79.00	63.49	3.5	64.00
	Total acquisition time (seconds)	65	1.00	167.00	45.82	33.2	36.00
	Total dose area product (Gy.cm^2^)	65	0.46	152.20	9.33	19.9	3.40
Plane B	kVp	65	60.00	105.00	64.29	6.1	64.00
	Total acquisition time (seconds)	65	1.00	167.00	46.95	34.2	37.00
	Total dose area product (Gy.cm^2^)	65	0.19	148.50	6.05	18.8	1.50
Total fluoro-time (seconds)		84	203.00	8,580.00	1,270.23	1,268.2	828.50

The mean difference between the left and right eye doses was significant (p = 0.034); however, the mean difference between the left and right thyroid doses was not significant (p = 0.281) (Table 3).

**Table 3:** Comparison between right and left doses for the eyes and thyroid.

Paired-samples statistics		N	Mean ± SD	Mean difference	P-value
Eye dose (mGy)	Right	89	0.08 ± 0.1	-0.018	0.034
	Left	89	0.10 ± 0.1		
Thyroid dose (mGy)	Right	88	0.08 ± 0.1	0.008	0.281
	Left	88	0.08 ± 0.1		

On correlating eye and thyroid doses with procedure types, both eye and thyroid doses were significantly correlated (p = 0.02 and p=0.009, respectively). Furthermore, eye and thyroid doses were not significantly correlated with patients’ age groups (p = 0.452), but thyroid dose was significantly correlated (p = 0.016) (Table 4).

**Table 4:** Correlating eye and thyroid doses with case type and age group.

Variables		Eye dose (mGy)	Thyroid dose (mGy)
Case	Not complicated	0.086 ± 0.047	0.063 ± 0.033
	Diagnostic	0.085 ± 0.034	0.079 ± 0.056
	Complicated	0.122 ± 0.068	0.116 ± 0.094
P-value		0.020	0.009
Age of Patient	<1 year	0.089 ± 0.049	0.081 ± 0.057
	1–5 years	0.085 ± 0.046	0.063 ± 0.030
	6–10 years	0.111 ± 0.063	0.103 ± 0.078
	11–14 years	0.102 ± 0.024	0.071 ± 0.029
	>14 years	0.112 ± 0.058	0.173 ± 0.182
P-value		0.452	0.016

The eye dose was significantly associated with the total fluoroscopy time (p < 0.001), although the thyroid dose was not (p = 0.544) (Table 5).

**Table 5:** Correlation between total fluoro-time and eye and thyroid doses.

Correlations		Eye dose	Thyroid dose
Total fluro-time (seconds)	r	0.426	0.069
	P-value	<0.001	0.544
	N	80	79

## Discussion

Pediatric interventional cardiologists perform both diagnostic and therapeutic procedures, exposing patients to radiation. In this prospective study, the mean eye dose measured was 0.09 mGy. In comparison, Sulieman et al. [2] conducted their study at four different sites using four different X-ray machines and models of therapeutic procedures, none of which were similar to the machines used in our study. They used medical adhesive tape to secure a dosimeter on the forehead to measure the eye lens doses. The mean eye lens dose measured by the cardiologists per procedure was higher (0.132 mGy) than that in our measurements. Sulieman et al. used an average tube voltage of 72 kVp, higher than the average tube voltage in our study (64.2 kVp). Additionally, the mean age of the pediatric patients in their study was 4.3 years, which was higher than that in our study (3.8 years). The higher tube voltage for the machine and the bigger size of pediatric patients and its effect on scatter radiation explains their higher measured eye lens doses compared to ours. Haga et al. [6] measured the cumulative eye doses over a six-month period. Their results showed a cumulative dose of 3.1 mGy with a measurement under lead glasses and a cumulative dose of 7.9 mGy measuring over lead glasses. However, our measured doses were higher than those measured by Shoshtary et al. [15], who performed adult procedures with a mean thyroid dose of 0.04 mGy which is lower compared to our result mean thyroid dose of 0.08 mGy. The authors also determined that the mean eye dose was 0.03 mGy which is also lower than our mean eye dose of 0.09 mGy. The procedures were performed using a Siemens system (Axiom Artis) with a 30-80 kVp tube voltage. In addition, lead glass screens were used below and above the procedure table, along with the proper wearing of thyroid collars and lead glasses. Moreover, the justification for this may be related to the difference in the procedural techniques established between adult and pediatric patients, as cardiologists tend to lean forward and remain closer during pediatric procedures [5].

Furthermore, a significant difference between the right and left eye doses was observed (p = 0.034). This can be attributed to variations in standing positions adopted by the cardiologists during the procedure, as well as the placement of the dosimeters above the eyes on the level of the eyebrows, which may yield dissimilar results when compared to studies where dosimeters are situated more laterally or centrally. On the other hand, the non-significant difference between the left and right thyroid sides is justified by how closely both dosimeters measuring the thyroid lobe doses are situated in comparison to the greater distance between the dosimeters measuring the eye dose, implying that scattered radiation reaching the thyroid will most likely affect both sides similarly.

In correlation of fluoro-time with doses, Suliman et al. [2] had a mean of 13 minutes per procedure compared to our study’s mean fluoro-time of 21 minutes per procedure. Their study’s measurement of eye lens doses was higher compared to ours. This could indicate that other parameters of the study such as their use of a different type of machine and its implementation of higher tube voltage have much more impact on the scatter dose exposure than the fluoro-time.

Different pediatric age groups have considerably different sizes, as explained by the rapid growth during that period. Our results showed a significant correlation between the thyroid dose and different patient age groups (p = 0.016). However, the eye dose showed no significant differences (p = 0.452). Perisinakis et al. [3] used a phantom to measure the scatter exposure of radiation at the eye and waist levels, which was correlated to many factors, including patient size. Their results showed that the scattered radiation around patients increased significantly with patient size. This clarifies the significance of our thyroid measurements by comparing the eye positions further away from the scatter area.

However, the correlation between different procedure categories and dose measurement showed that higher doses were recorded for complicated procedures compared to non-complicated and diagnostic procedures, indicating the influence of longer procedure time, complexity, and usage of multiple techniques in complicated cases on the radiation absorbed by the cardiologist. Furthermore, in non-complicated procedures, it may be easier to employ protective measures effectively, such as minimizing the use of fluoroscopy or maintaining a greater distance from the radiation source. In complicated cases, these measures may be more challenging to implement without compromising patient safety or procedural success. Our study showed significant correlations between the eye and thyroid doses according to the type of procedure performed (Table 4). This is similar to the results of Efstathopoulos et al. [16], who measured the eye dose and other dosimeters for the wrists and lower extremities of adult interventional cardiologists using a monoplane machine. Their results showed that, for particular interventional procedures, physicians were subjected to higher doses of scatter radiation, with relatively higher doses absorbed in pacemaker implantations than in diagnostic coronary angiography. Furthermore, the mean eye dose for cardiologists in their study was 0.013 mGy, which is lower than our results. However, they did not investigate thyroid dose measurements.

Several factors may have contributed to the variations in the absorbed doses between studies. These factors include how cardiologists utilize their protective equipment, the specific type and tube voltage of the X-ray machines employed, variations in the complexity of the procedures performed, the alternating positioning of the dosimeters for eye or thyroid measurements, and their placement during the procedure.

## Conclusions

The current study measured occupational doses to the eyes and thyroid of pediatric interventional cardiologists during different procedures in a catheterization lab. A significant amount of radiation was measured in both organs, leading to higher doses of radiation than those recommended by the International Commission on Radiological Protection. Eye dose measurements should be considered when reviewing staff’s occupational doses annually. In addition, radiation dose measurements varied between different types of procedures. Our results indicate the importance and necessity of applying the radiation protection concept of dose optimization, including establishing diagnostic reference levels for different protocols.
